# Sleep, Wake, and Critical Brain States: Corollaries From Brain Dynamics

**DOI:** 10.3389/fnins.2018.00948

**Published:** 2018-12-11

**Authors:** Kartik K. Iyer

**Affiliations:** ^1^UQ Centre for Clinical Research, Faculty of Medicine, The University of Queensland, Brisbane, QLD, Australia; ^2^UQ Child Health Research Centre, Faculty of Medicine, The University of Queensland, Brisbane, QLD, Australia; ^3^Department of Biological Sciences, Faculty of Science, University of Western Australia, Perth, WA, Australia

**Keywords:** sleep wake, cortical activity, neuronal firing, EEG, LFP, criticality

A balanced sleep-wake cycle is essential for maintaining an optimum circadian rhythm, as this feature of mammalian systems largely determines our overall alertness, cognition, and general well-being. When our circadian rhythm is disrupted by prolonged periods of wakefulness or hypersomnolence, our brain's “master clock”—the suprachiasmatic nucleus (SCN)—triggers spontaneous neuronal firing patterns (Scammell et al., 2017). The spontaneous nature of these firing patterns directly affects activity within large-scale cortical networks which influence our ability to process information and optimize function during wake periods. Changes to neuronal firing patterns are thought to be reflected by transitions in spatial and temporal fluctuations in cortical activity that subscribe to the hypothesis that the brain operates within critical states, i.e., states where the balance in neuronal processes are either maintained or transformed. Such hypotheses of critical brain states have rapidly become an established theoretic basis for distinguishing healthy and pathological brain activity (Cocchi et al., 2017). Studies that better characterize the rich spatiotemporal properties of cortical activity during critical brain states with a focus on sleep-wake cycles therefore substantiate our understanding of how the mammalian brain optimizes information processing and reorganizes brain networks during alert, fatigued and sleep states. Hypothesis-driven studies within these dynamic regimes of brain activity have far reaching implications for understanding human cognition, behavior, and overall health.

## Critical Brain States During Sleep and Wake Periods

Current studies of critical brain states have sought to detail changes occurring within slow and fast timescales of cortical activity present during vigilance states of wakefulness, fatigue, and recovery periods of sleep. Seemingly, the most salient aspect of cortical activity as a function of time awake is the decline in long-range temporal correlations (LRTC's) within brain networks. These LRTC's seek to explicate the scale-invariant or *self-similar* nature of neuronal oscillations occurring in cortical activity known as 1/f noise. The pervasive nature of these cortical activity timescales have been demonstrated to increase in signal-to-noise ratio during information processing and optimization e.g., working memory tasks and decision-making (Linkenkaer-Hansen et al., 2001; Friston et al., 2012; Meisel et al., 2013; Kringelbach et al., 2015). Therefore, the characterization of short and long timescales of cortical activity across wake, extended wake and sleep periods allows us to examine unique spatiotemporal properties present in large-scale networks responsible for cognitive and behavioral performance. Such properties are also essential in probing how the brain's dependency on sleep purportedly causes shifts away from these critical brain states to subcritical (or less efficient) brain states.

It has been a long-held view that critical brain states generally underpins scale-free cortical activity, i.e., small or large fluctuating timescales of activity that are highly ordered despite their seemingly random occurrences (He et al., 2010). Scale-free regimes of brain activity provide an insight into system disturbances, which potentially arise from a balance between excitation and inhibition in cortical pathways and, more broadly, the complex firing dynamics of neural masses present within neuronal networks. In mammalian brains it has been demonstrated that cortical networks exhibit these scale-free critical states of brain activity when awake from a sleep period, and as a function of time awake, slowly fade away from critical behavior during prolonged wake periods (Meisel et al., 2013). Cortical activity timescales that shift away from critical states, e.g., during extended wakefulness, are more representative of a subcritical brain state. These brain states tend to denote cortical activity that is less ordered and irregular in occurrence, conforming to multiple timescale characteristics (i.e., mixed durations of activity). Subcritical regimes of cortical activity are thought to feature strongly during specific vigilance and sleep states (e.g., NREM or extended wakefulness) where cortical network activity undergoes a complex switching of dynamic states and may contribute to providing the mechanism by which cortical timescales are restored to operate within critical regimes during wake periods (Meisel et al., 2013; Priesemann et al., 2013).

## The “Bottom-Up” Influences to Critical Brain States of Sleep and Wake

Animal and computational modeling approaches have further explored on mechanisms which influence critical states of cortical activity timescales in segregated functions of wake, fatigue, and recovery sleep periods. What we know now is that within sleep, distinct modes of neuronal network behavior in non-rapid eye movement (NREM) and rapid eye movement (REM) sleep are at play (Vyazovskiy et al., 2009; Scammell et al., 2017). A considerable advancement in our understanding, however, is the characterization of cortical activity during sleep and wake to better elucidate timescale changes in neuronal firing (spiking) patterns present during these periodic cycles. It has been demonstrated in rodent and computational studies that NREM sleep and sleep deprivation have periods of “offline” neuronal firing (Vyazovskiy et al., 2011) resulting in a stereotypical occurrence of low frequency, intermittent large amplitude events in local field potentials (LFPs) (Meisel et al., 2017a). As NREM periods progressed, gradual increases in neuronal firing patterns returned, correlating with a re-emergence of slow autocorrelation decay functions in LFP data (Meisel et al., 2017a) and a return to a critical regime of activity (Meisel et al., 2013). What is particularly encouraging is that a comparative, follow-up human electroencephalography (EEG) study by Meisel and colleagues (Meisel et al., 2017b) confirms this cyclical switch in cortical activity timescales during these sleep and wake states.

A combination of several mechanisms could, ostensibly, contribute toward these transitions between critical and subcritical brain states during sleep and wake states. In sleep states, the inhibition and disinhibition of cholinergic and aminergic neurotransmitters during NREM and REM sleep, via sleep promoting nuclei in brainstem areas and release of gamma-Aminobutyric acid (GABA), has been associated with increases in sleep spindles ranging between delta and alpha frequencies in EEG (Deboer et al., 2003; Wulff et al., 2010). Projecting pathways between brainstem and cortex—more formally known as the ascending reticular activating system (ARAS)—are also key in linking complex synaptic relays between deep brain nuclei to firing rates within populations of neural masses. In states of wakefulness, the mediation of conscious states via ARAS and interplay of metabolic demand [e.g., breakdown of adenosine triphosphate (ATP)], are in a way, a rate determining step in the way these neuronal firing patterns are scaled over the function of wakefulness, and more pointedly, in our capability to optimize processing of cognitive information.

In this context, the role of sleep and wake states in cortical and subcortical areas (Mitra et al., 2015; Ribeiro et al., 2016; Daffertshofer et al., 2018; Gaggioni et al., 2018) have also provided emerging details in differential scales of brain activity during vigilance states and broader changes to whole-brain connectivity. To extend on previous points, transitions away from critical states could be influenced by changes occurring from subcortical structures such as the brainstem, hypothalamus and basal forebrain via neuromodulation (Jones, 2005) or influenced by activity-dependent synaptic strengths (Tononi and Cirelli, 2014; Kuhn et al., 2016; De Vivo et al., 2017). From a large-scale brain network level, functional and effective connectivity during deeper periods of NREM sleep may potentially involve unique optimization processes (e.g., pruning of connections) between distinct neural regions and contribute to the breakdown in causal interactions (Massimini et al., 2005; Jobst et al., 2017; Tagliazucchi and Van Someren, 2017). As these NREM periods progress and neuronal firing becomes more frequent, there is a cumulative increase in scale-invariance between anatomical and functional networks. Thus, revisiting the thematic link between critical, subcritical, and near-critical brain states in sleep-wake cycles with whole-brain connectivity changes in brain function and anatomy is an encouraging hypothesis-driven avenue for assessing and characterizing changes in cognitive performance and/or pathological brain states.

## Avenues TOWARD Optimizing Spatiotemporal Metrics in Sleep and Wake Functions

For neurophysiologists and electrophysiologists alike, characteristic cortical activity profiles present in sleep and rest periods (e.g., low and high frequency events in LFP or EEG data), within the purview of critical brain states, is an immediate area in which human behavior and pathological brain activity is primed for further study. Whole-brain functional connectivity studies have offered a view into putative correlations that exists within and between brain regions in the sleep-wake cycle. However, these approaches have proven limitation in their lack of specificity of more complex sleep phenomena, such as sleep disorders (e.g., insomnia) and the effects of consciousness and awareness (Tagliazucchi and Van Someren, 2017). In this regard, we are yet to establish whether specific temporal profiles of brain activity within critical brain states of sleep and wake can complement current network driven approaches and potentially enable better characterization of typical vs. atypical brain response. Detailing the transitions of these temporal snapshots across the brain, as a function of sleep and wake, could neatly build on the hypothesis that spontaneous neuronal activity conforms to a critical state and exhibits universally scaled dynamics (Beggs and Plenz, 2003).

**Figure 1:**
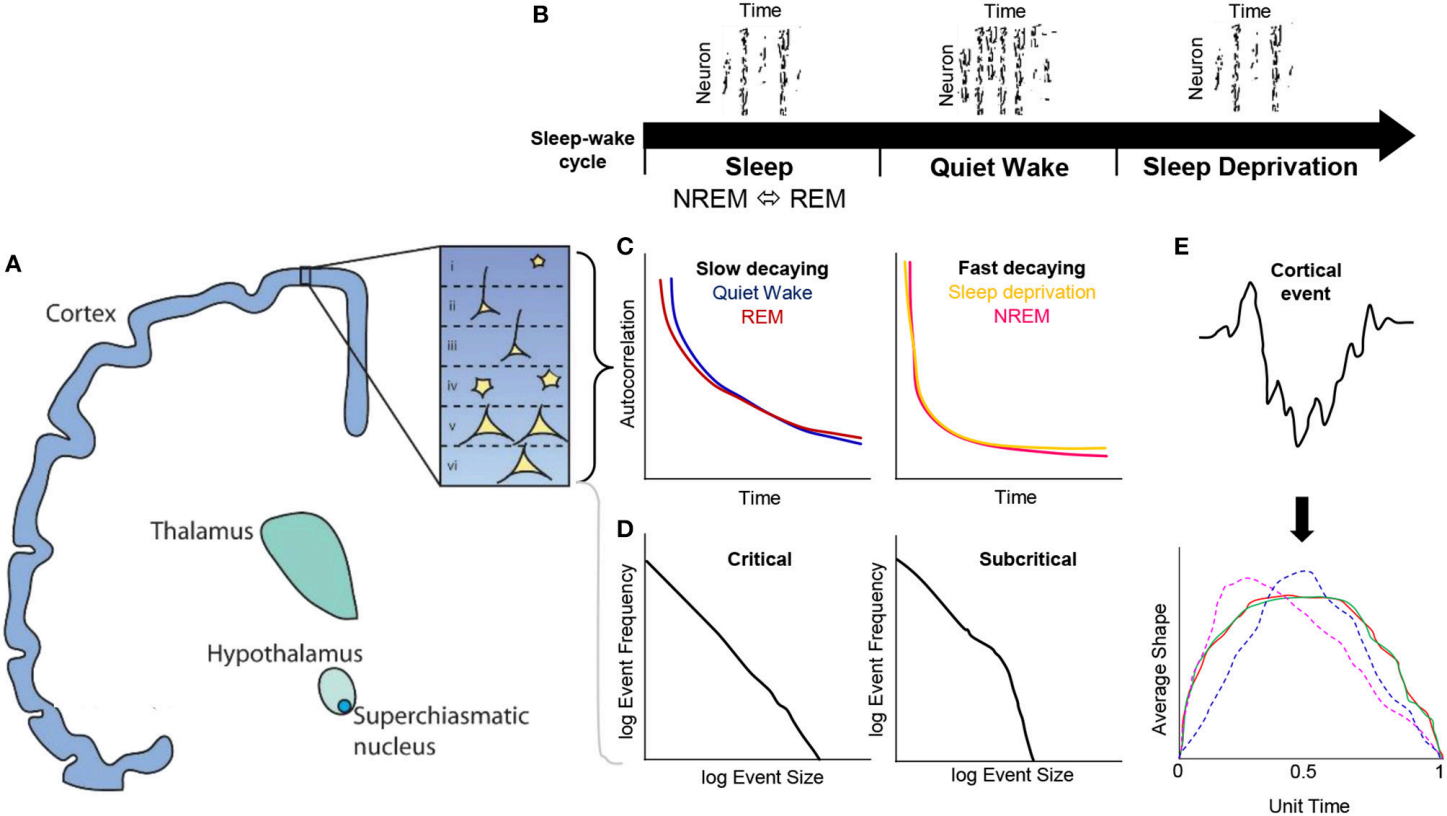
Conceptual overview of cortical dynamics present during the sleep-wake cycle in the context of critical brain states. **(A)** Ascending pathways from the suprachiasmatic nucleus, hypothalamus, thalamus to cortex, where layers I—VI have been recently explored with respect to cortical dynamics. **(B)** The presence of offline periods of neuronal firing during NREM sleep and sleep deprivation. **(C)** Temporal autocorrelations of neuron firing reveal that slowly decaying functions are present during quiet wake and REM sleep, whereas fast decaying rates were found in NREM and sleep deprivation vigilance states. **(D)** Probability distributions have been used to characterize event scales vs. their frequency, e.g., neurons fired per second vs. log of neurons fired frequency. These distributions when tested against model likelihood functions such as power-laws, revealing universal scaling relationships that sits within a critical or subcritical regime. **(E)** Cortical activity can also be further characterized by partitioning fluctuations (e.g., EEG or LFP amplitudes) into a fixed hierarchy of duration bins and averaging these amplitudes to derive an average shape. These average shapes can be further renormalized to have a unit time and unit area to directly compare short and long time scales of cortical activity, e.g., average shapes at different durations that collapse on one another (red, green) indicate scaling functions consistent with criticality; however, if these average shapes differ from each other at different durations (magenta, blue) they may indicate deviations away from a critical system, an important consideration for addressing mechanisms of neuron firing and spatiotemporal activity during NREM, REM, quiet wake and sleep deprivation periods.

In mammalian studies, temporally-rich methods do offer an avenue for exploring this further. Averaged, scale-invariant profiles of cortical fluctuations known as: “neuronal avalanche shape” (Friedman et al., 2012) *or* “cortical burst shape” (Roberts et al., 2014), respectively, represent one such technique. These methods build upon neuronal network approaches to provide a direct comparability how averaged shapes of neuronal firing or amplitude fluctuations change between short and long duration bins. Indeed, the emergence of these techniques are shining a light on transitions in critical brain states during rest and sleep under anesthesia (Fekete et al., 2018). As such, they provide promise for neuroscientists in characterizing the dynamic range of activity that exists within sleep and rest periods of EEG or LFP data. Applications of averaged shapes have had vast implications in characterizing properties of physical phenomena (e.g., ferromagnetic materials), where average event shapes have proven to conform to universal scaling functions during critical states (Sethna et al., 2001; Papanikolaou et al., 2011; Gleeson and Durrett, 2017), allowing for better inferences into self-organization processes that occur within physical systems. Average neuronal avalanche shapes and their universal scaling functions thus allow us to refine on the narrative that critical (and subcritical) behavior of cortical activity may be inextricably linked with biological self-organization, such as the maintenance of complex metabolic processes and homeostatic regulation. See summary Figure 1 for a conceptual overview.

In terms of the broader neuroscientific picture, probing the dynamics of cortical activity within these critical brain state regimes allow for an optimized study of large-scale transitions of cortical activity existing within sleep and resting states, which can be measured against behavioral performance including cognitive impairment and other deficits such as memory and attention. These are emergent approaches that are beginning to indicate potential differences in the dynamic range of cortical activity that exists between quiet rest (near-critical states) and focused cognitive attention (Fagerholm et al., 2015). Identifying declines in cortical timescales during sleep and wake cycles in mammalian models thus presents a major step toward understanding the neuronal correlates in human subjects with noisy, granular datasets such as those acquired by EEG, MEG, and fMRI methods. Further integration of the findings and techniques discussed offers promising avenues for data rich interpretation of the sleep-wake cycle and its intrinsic relationship to regulating the cortical dynamics of brain behavior.

## Author Contributions

This is a sole author paper by KKI who is responsible for the synthesis of prior work to discuss concepts and techniques related to the subject matter.

### Conflict of Interest Statement

The author declares that the research was conducted in the absence of any commercial or financial relationships that could be construed as a potential conflict of interest.
